# An in-depth investigation of the impact of salt nature on the formulation of microemulsion systems

**DOI:** 10.1038/s41598-023-40761-x

**Published:** 2023-09-01

**Authors:** Ali Rezaie, Hassan Ghasemi, Fatemeh Eslami

**Affiliations:** Department of Chemical Engineering, Tarbiat Modares University, Jalal Al Ahmad HWY, P.O. Box: 14115-111, Tehran, Iran

**Keywords:** Chemical engineering, Chemical physics

## Abstract

Electrolytes have a wide range of technological applications. Despite the recent improvements in characterizing and predicting the phase behavior of microemulsion systems by hydrophilic-lipophilic deviation (HLD) and net-average curvature (NAC) frameworks, they are ineffective in the presence of different salts. This work seeks to bridge this gap by investigating the influence of salt nature on the microemulsion phase formulation. First, a one-dimensional salinity scan on different microemulsion systems consisting of sodium dodecyl benzene sulfonate as a surfactant, hexane as an oil and, several brines was carried out, and the effect of each salt on the phase behavior were precisely evaluated. The results for optimum salinity and solubilization parameter of different salts were consistent with the Hofmeister series. In addition, multiple linear regression model is presented to accurately predicting the optimum salinity of different salts using this research data and all the available experimental data. The results revealed that the values estimated by this model is in significant consistency with the experimental data by correlation coefficient of 0.92. Finally, the effect of salt type on the NAC parameters (length parameter, and characteristic length$$)$$ were evaluated to improve the predicting ability of this equation of state in the presence of various salts. We found that salt nature has a significant impact on both these parameters. It was found that the length parameter is linearly dependent on the optimum ionic strength of salts while the salting-out capacity of each salt was predominant factor affecting the characteristic length.

## Introduction

Microemulsions are thermodynamically stable and optically transparent/translucent surfactant–oil–water (SOW) systems^1^. Four types of microemulsion systems form with changes in formulation variables, Winsor type I, II, III and IV. In Winsor I systems, an excess oil phase and an O/W microemulsion which consists of oil-swollen micelles are in equilibrium^2,3^. Among Winsor III microemulsions, the optimum formulation is the one where the amounts of solubilized oil and water are equal and at the maximum amount. At this optimum point, a deep fall happens in the interfacial tension (IFT), resulting in maximum oil/water separation and enhanced oil recovery^4^.

Salts are crucial ingredients in formulating the microemulsions and considerably affect the phase behavior and optimum formulation of SOW systems^5^. As a result, understanding the behavior and predicting the extent of the impact of different salts on the phase behavior and properties of microemulsions gives profound insight to design SOW systems such as crude oil dewatering, enhanced oil recovery, drug delivery, nanoparticles and cleaning products synthesis^6–8^. It has been demonstrated that O/W microemulsions can be utilized to synthesize a wide range of nanomaterials with controlled characteristics^9,10^. O/W microemulsions are used as potential delivery systems since they increase lipophilic drugs' water solubility and absorption rate^11,12^.

In this respect, Hofmeister ordered ions according to their salting-out capacity as follows:

$${Al}^{3+}> {Mg}^{2+} > {Ca}^{2+}>{Li}^{+} >{Na}^{+}>{K}^{+}>{NH}_{4}^{+}$$

$${CO}_{3}^{2-}> {SO}_{4}^{2-} > {F}^{-}>{Cl}^{-} >{Br}^{-}>{NO}_{3}^{-}>{I}^{-}$$

Salting-out ions (left hand side) which usually have a larger hydration radius, lowers the solubility of other solutes such as surfactants. From left to right, the salting-out power of ions decreases and at the extreme conditions, this power may be inversed. In this situation, ions may even improve the solute solubility and show the salting-in behavior. In general, the left-sided and right-sided ions in Hofmeister series are known as salting-out and salting-in ions^13–15^. It has been suggested that charge density and hydration radius of ions are important parameters that govern whether ions are salting-out or salting-in.

According to Liu et al., the presence of cations causes interfacial dehydration of solutes, and micelle transition originates from hydration competition between ions and surfactants. Zavitsas reported that salting-out behavior of ions is due to their water-binding capacity, which is in direct relation with charge density of ions. In addition, Liu provided some evidence that there is a linear relationship between the charge density of ions and their optimum salinity^16–19^.

Regarding the salting out phenomenon, some researchers have conducted a variety of experiments to acquire further information about the impact of ions on the phase behavior properties of SOW systems. Accordingly, it can be said that there is strong connection between the nature and concentration of ions and the solubilization capacity of surfactant in SOW systems^20^. The latter can be quantified by the solubilization parameter (SP) which is the ability of a surfactant to solubilize oil and water in the microemulsion phase^5^.

Many researchers have tried to develop a mathematical framework to quantify and predict the phase behavior of SOW systems. Salager and colleagues introduced the semi-empirical hydrophilic-lipophilic deviation (HLD) equation to address this issue. The positive, negative, and zero HLD values reflect Winsor type I, II, and III, respectively^21–23^. For ionic systems, the HLD equation is presented as:

1 $$HLD=\mathrm{ln}\left(S\right)-k\left(EACN\right)- {a}_{T}\left(\Delta T\right)-f\left(A\right)+ {C}_{c}$$

In this expression, S represents the salinity of the aqueous phase, EACN is the oil's hydrophilic character. The parameter ${C}_{c}$ is the surfactant's characteristic curvature, which represents the surfactant's hydrophilic-lipophilic nature^24,25^. The term f(A) is an indicator of the type and concentration of cosurfactants in the system. In the absence of any cosurfactant, this parameter is considered to be zero^26,27^. The temperature deviation from the reference temperature (25 °C) is known as $\Delta T$^28^. ${a}_{T}$ is temperature constant and for ionic surfactants equals to 0.01 ${K}^{-1}.$ Although ion’s nature significantly affects the salinity term, they are not explicitly incorporated in the HLD equation. Therefore, presenting a correlation to predict the optimum salinity of various salts is crucial. Anton and Salager were the first to propose this equation:

2 $${\mathrm{S}}_{\mathrm{Ne}}= \frac{2}{1+\mathrm{Z}}\times {\mathrm{S}}_{\mathrm{N}}$$

where Z is the anion valence, ${\mathrm{S}}_{\mathrm{N}}$ is the normality of sodium salt, and ${\mathrm{S}}_{\mathrm{Ne}}$ is the equivalent optimum salinity of sodium salt. It is obvious that this equation is only applicable to sodium salts^29^.

Warren and Harwell added a hydration number term to the Eq. (2), as follows:

3 $$S= {S}^{*} \times \frac{{M}_{{W}_{NaCl}}}{{M}_{{W}_{salt}}} \times \frac{2}{1+Z} \times \mathrm{ln}( {h}_{c} )$$

where ${S}^{*}$, *M*_*W*_ and ℎ*c* are the optimum salinity of sodium chloride obtained by the HLD equation, molecular weight and hydration number of ions, respectively. The lack of a unique methodology to measure the hydration number of ions and overestimation of the optimum salinity of various salts are the major problems of this equation^30,31^.

On the other hand, the NAC model was developed by Acosta et al. to upgrade the applicability of HLD by predicting the properties of SOW systems. The NAC model is based on two statistical descriptions^26^. These equations are:

4 $$ H_{n} = \left( { \frac{1}{{R_{o} }} {-} \frac{1}{{R_{w} }} } \right) = - \frac{HLD}{L} $$

5 $${H}_{a}= \frac{1}{2} \left( \frac{1}{{R}_{o}}+ \frac{1}{{R}_{w}} \right) \ge \frac{1}{\xi }$$

${H}_{n}$, ${H}_{a}$, L and $\xi$ are net curvature, average curvature, length parameter and characteristic length, respectively. ${H}_{n}$ is positive for Winsor I, near to zero in bi-continuous systems (zero for optimum salinity), and negative for Winsor II. The phase behavior switches from Winsor I to Winsor III to Winsor II as a result of the change in net-curvature from positive to zero to negative^32^. ${R}_{o}$ and ${R}_{w}$ represent the radii of coexisting hypothetical spherical aggregates of oil and water in microemulsion phase^26^. The length parameter is a measure of surfactant hydrophobicity. It is proportional to the extended length of the surfactant tail group, which is a function of linear carbon number in the surfactant tail^33–35^.

Characteristic length quantifies the interaction power of surfactant molecules at the interface with the oil/water molecules in the bulk phase. The farther the surfactant molecule can extend its influence, the higher the characteristic length value will be. Both L and $\xi$ are fitting parameters and are calculated by salinity scans^4,27^.

Despite the advantages provided by the NAC model, it cannot predict the properties of microemulsion systems containing salts other than NaCl.

To fulfill this shortcoming, we designed the present study to evaluate the salt effect on the phase behavior of SOW systems. In this regard, we propose a new robust and convenient correlation to predict the optimum salinity of various salts based on the formulation variables and intrinsic properties of salts. We believe this correlation is more precise and easier to use in comparison with the similar ones. Therefore, a one-dimensional salinity scan was performed for a series of SOW system containing sodium dodecyl benzene sulfonate (SDBS), hexane and the following salts: $\mathrm{NaCl}$, $\mathrm{KCl}$, ${\mathrm{MgCl}}_{2}$, ${\mathrm{CaCl}}_{2}$,${\mathrm{ NaNO}}_{3}$, ${\mathrm{Na}}_{2}{\mathrm{SO}}_{4}$, ${\mathrm{Na}}_{2}{\mathrm{CO}}_{3}$.

In addition, we investigate the effect of various salts on the NAC model parameters ($L, \xi$) to predict the microemulsions properties, more accurately.

## Materials and methods

### Materials

SDBS was purchased from Sigma Aldrich, and used as the anionic surfactant. The oil was the analytical-grade n-hexane (${C}_{6}{H}_{14}$) purchased from Chem-lab. Salts used in the study include sodium chloride ($\mathrm{NaCl}$), potassium chloride ($\mathrm{KCl}$), magnesium chloride (${\mathrm{MgCl}}_{2 })$, calcium chloride (${\mathrm{CaCl}}_{2}$), sodium nitrate (${\mathrm{NaNO}}_{3}$), sodium sulfate (${\mathrm{Na}}_{2}{\mathrm{SO}}_{4}$), and sodium carbonate (${\mathrm{Na}}_{2}{\mathrm{CO}}_{3}$), all of which purchased from Sigma Aldrich. To avoid deliquescence, we maintained all these electrolytes in the dryers. Distilled water was purchased from Merck. Without any additional purification, all compounds were used as supplied.

### Methods

#### Salinity scan

Salinity scan experiments were performed by mixing 5 mL of hexane and 5 mL of aqueous solution in 15 mL graduated test tubes. Before adding the aqueous phase to the test tubes, the components (surfactant, water and salts) were well mixed together by stirring for three minutes. The surfactant concentration in aqueous phase was constant, at 2 $\mathrm{w}/\mathrm{v} \%$ of system (oil phase + aqueous phase). At water oil ratio (WOR) = 1 and room temperature (25 $\pm$ 2 °C), different salts were utilized separately to evaluate their impact on the phase behavior of SOW systems. Salinity scans were conducted by changing the salt concentration in aqueous solution ($(1-10\,g\,salt\,/\,100\,mL\,of\,aqueous\,phase)$). All vials containing ingredients were gently hand-shaken 20 times, and then allowed to equilibrate for 24 h to reach microemulsions.

Previously, two methods have been presented for the visual selection of the optimum salinity tube (S*)^36,37^. The first method chooses the tube with equal excess phase volumes as the optimum salinity. The second method selects the tube that shows the fastest phase separation. In this work, we use the first method to find the S* tube and apply the second method when the first condition is not met.

#### Fitting NAC parameters

The NAC parameters are fitted by the phase volume fraction data based on the method proposed in previous works^27,33^. In this regard, after the phases in graduated test tubes reached equilibrium, the experimental upper and lower boundaries of each tube were determined by measuring the levels of each phase in the test tube. Then, the corresponding HLD values of test tubes and surfactant parameters such as molecular weight and head area and phase volume fractions were introduced to the HLD-NAC equation of state. Finally, the objective function was minimized by Matlab and NAC parameters were obtained. In the supplementary information file S1, we have provided a detailed explanation of fitting NAC parameters and HLD-NAC calculations. It is important to note that the salinity scans were repeated three times to ensure the accuracy and repeatability of the experimental data.

## Results

### Effect of different salts on the phase behavior of SDBS-Hexane-Brine system

**Figure 1:**
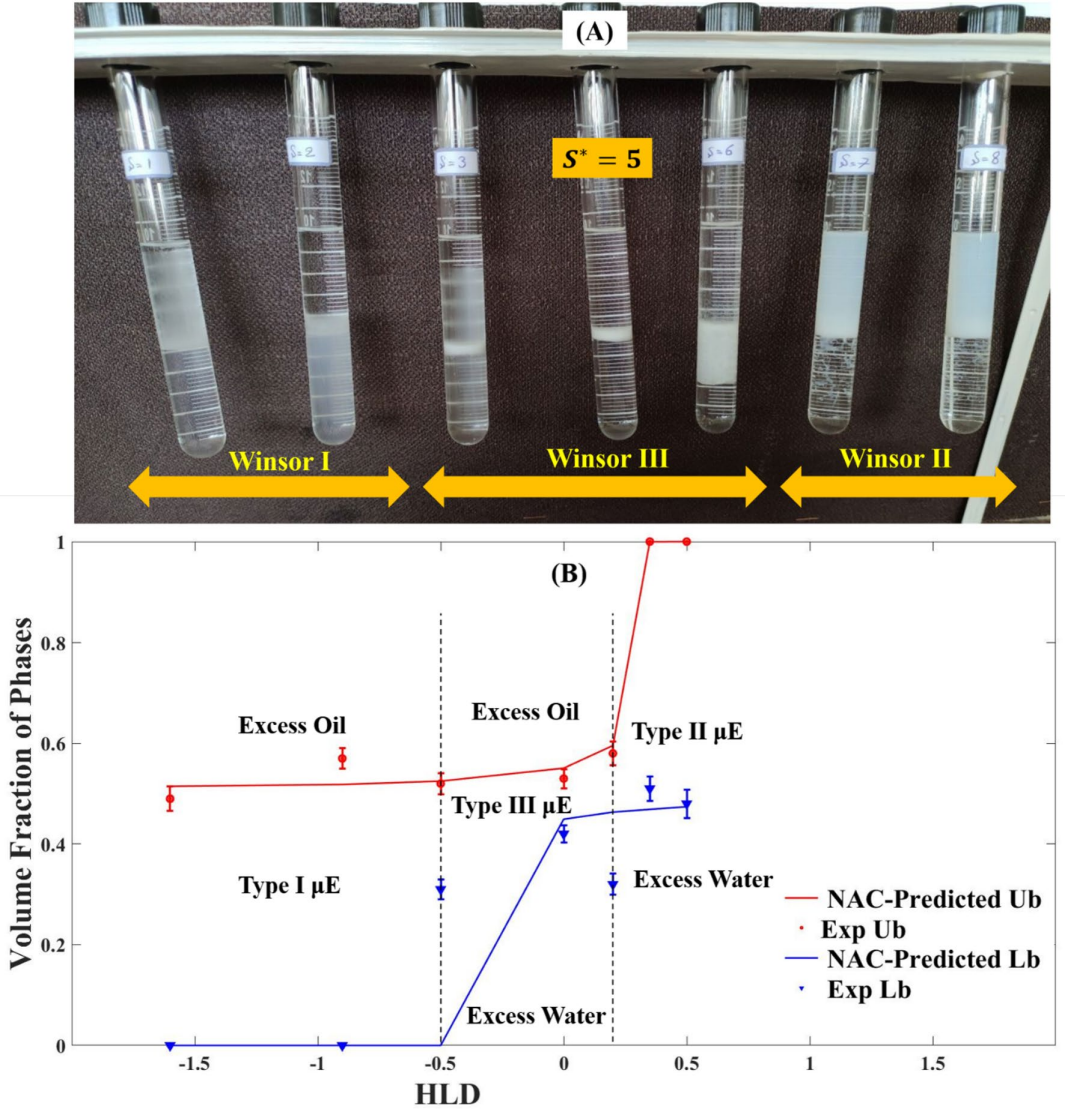
(**A**) Salinity Scan for a SDBS-hexane-potassium chloride system at $25^\circ \mathrm{C}$, WOR = 1 and constant SDBS concentration in system (2 ${w}/{v} \;\%$), The corresponding HLD values of each tube from left to right are − 1.6, − 0.9, − 0.5, 0, 0.2, 0.35, 0.5. (**B**) Comparison between Experiment and NAC-predicted phase volume fractions of excess oil, excess water and microemulsion phases in SDBS-hexane-potassium chloride as a function of HLD.

Salinity scans were conducted to examine how different salts affect the phase behavior of the SDBS–hexane–electrolyte system. As shown in Fig. 1A, the criterion of maximum and equal solubilization of water and oil in microemulsions was founded to be fulfilled at the optimum salinity point (S* = 5 $\mathrm{g\,KCl}\,/\,100\mathrm{\,mL\,of\,aqueous\,solution}$)^38^. Figure 1B illustrates the experimentally determined phase volume fractions of microemulsion in the presence of potassium chloride as salt and its estimated phase volume fractions (Ub and Lb) based on the NAC model. There is good agreement between the experiment and NAC-predicted phase volume percentages for most points. The Winsor I to III phase transition point shows the largest deviation: HLD-NAC predicts it to occur at HLD = − 0.9, while it actually happens at HLD = − 0.5. This means a 0.4 unit difference in HLD units. This deviation is acceptable compared to the literature^25,39^. Potassium chloride, which is not accounted for in the HLD equation, may cause this discrepancy. However, HLD and HLD-NAC can still adequately predict the actual phase behavior. Providing additional information about salinity scans of other salts, supplementary file S1 also describes the process for predicting the phase volume fractions of microemulsion systems using surfactant properties in Table. S1.

**Figure 2:**
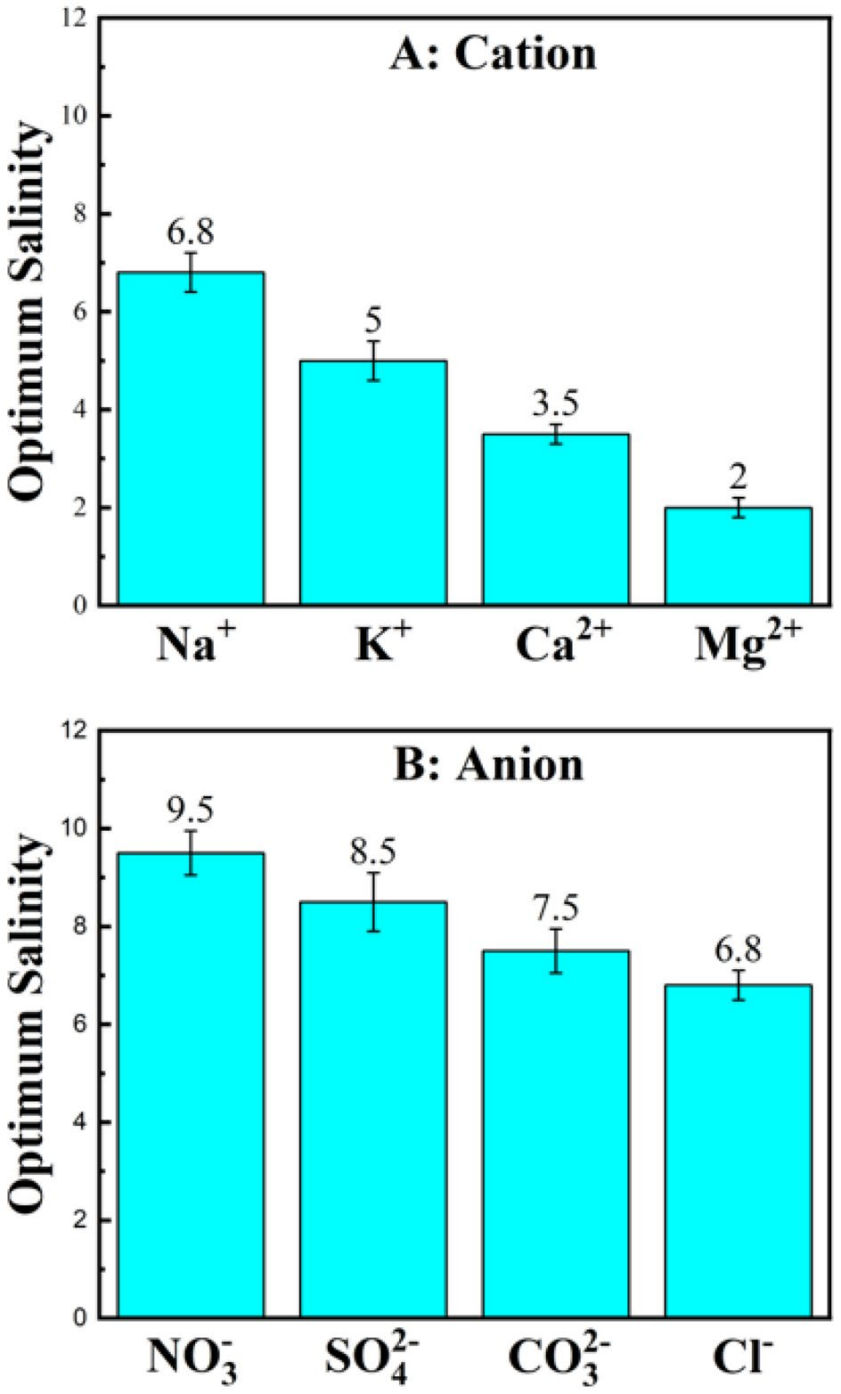
Effect of ions on optimum salinity of SDBS-Hexane-Electrolyte system. (**A**) Effect of cations from chloride salts. (**B**) Effect of anions from sodium salts.

The optimal salinity value of different salts is depicted in Fig. 2 for two kinds of anionic and cationic salts. The data illustrated in Fig. 2 shows that the salting-out ions (${\mathrm{Mg}}^{2+}$, ${\mathrm{Ca}}^{2+}$, ${\mathrm{CO}}_{3}^{2-}$, ${\mathrm{SO}}_{4}^{2-}$) produce phase inversion points at lower salt concentrations, whereas systems containing salting-in ions (${\mathrm{K}}^{+}$, ${\mathrm{NO}}_{3}^{-}$) have a higher optimal salinity value. This behavior can be interpreted based on the surfactant solubility variations in terms of Hofmeister ranking concepts^5,26^. Therefore, the lower the surfactant solubility in water, the sooner the phase transition happens from Winsor I → III → II in the salinity scan. Although the overall trend in Fig. 2 is in consistence with Hofmeister series, Na^+^ and Cl^-^ are the exceptions. Such a reversal trend in the Hofmeister series has been reported several times in the literature, but a clear justification has not been provided yet^40–45^. Sögaard et al. reported a reversal trend when divalent ions interacted with silica nanoparticles^46^. Oechsle et al. reported that the viscosity of collagen in the presence of different salts had a reversal trend according to the Hofmeister series^47^. Vera et al. observed a reversal between nitrate and chloride anions, while examining the solubility of surfactants in aqueous solutions^5^.

In addition, it has been demonstrated that salting-out ions decrease the micelle curvature while salting-in ions increase it. The reason for this phenomenon is attributed to the stronger electrostatic screening of salting-out ions, which reduces the repulsion between negatively charged micelles^48,49^. Therefore, it is proven that in the presence of salting-out ions, the optimum salinity, which is equal to net-zero-curvature point, moves to the lower values. For example, it is observed that the lowest optimum salinity belongs to the salt with the highest salting-out power, magnesium chloride.

**Figure 3:**
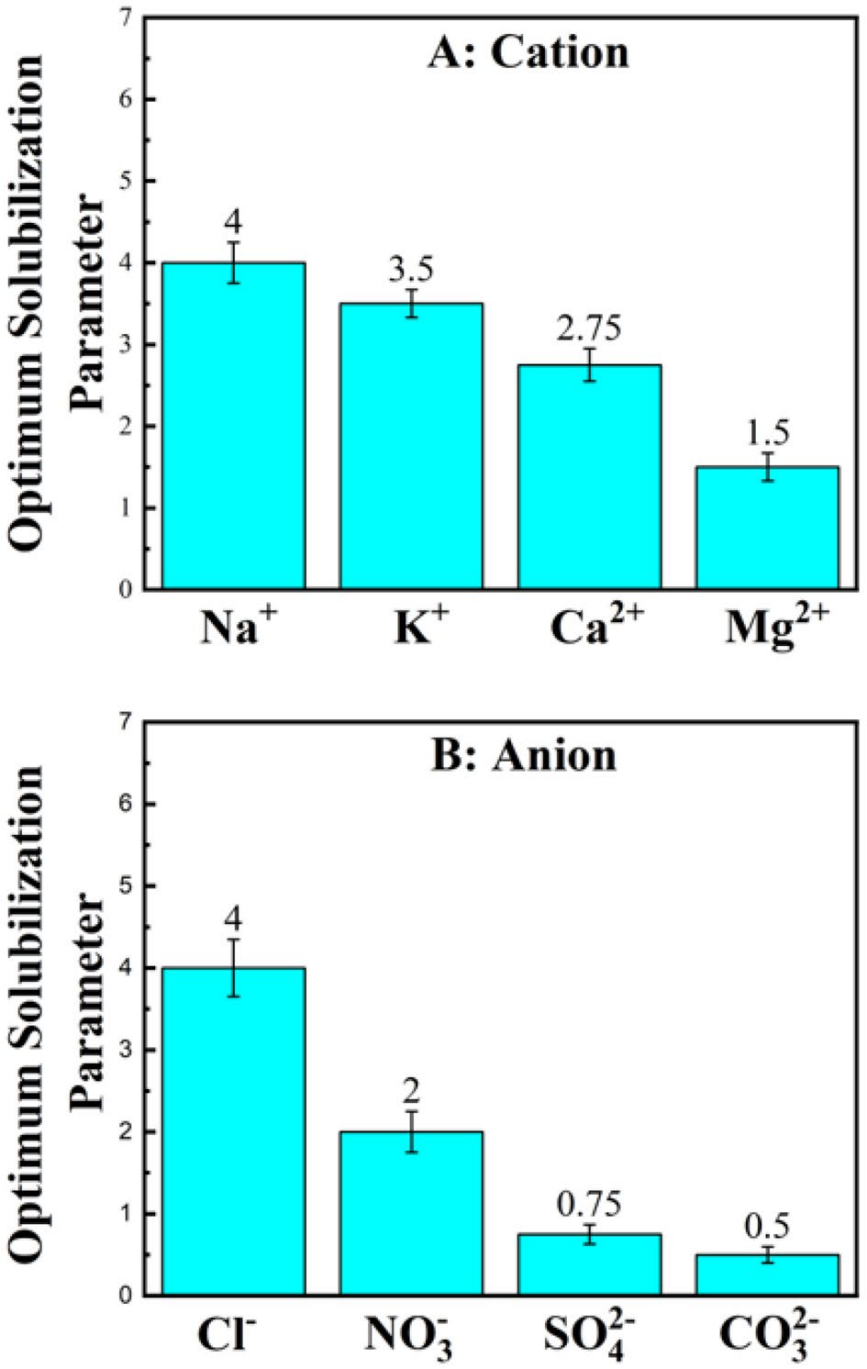
Effect of ions on optimum solubilization parameter of SDBS-Hexane-Electrolyte*. (A*) Effect of cations from chloride salts*. (B*) Effect of anions from sodium salts.

Figure 3 illustrates the effect of salt type on the optimum solubilization parameter of SDBS ${(SP}^{*})$ which is calculated as follows:

6 $${SP}^{*}=\frac{ml\, of\, water\, \left( or\, oil \right) in\, optimum\, microemulsion\, phase}{ml\, of\, surfactant\, in\, the\, system}$$

As can be seen in this figure, salting-in ions enhance the surfactant solubilization ability while salting-out ions reduce it being consistent with previous results^5,19^.

### A correlation for optimum salinity of various salts

Here we used the multiple linear regression (MLR) tool of Matlab software to develop a relationship between the optimal salinity values of different salts obtained experimentally from both the literature and this work, and the influential variables, affecting the optimum salinity of each salt. The target data are the optimum salinity ratios of different salts to that of sodium chloride^50–52^. optimum salinity of sodium chloride is calculated by substituting the formulation variables into the HLD equation. The input data contains 17 samples and 3 features. Influential characteristics on the salting-out behavior of salts were chosen as the feature to improve the prediction capability of correlation. The features are the charge density ratio of cation $$({X}_{1})$$, hydration thickness ratio of cation ($${X}_{2}$$) and molecular weight ratio of salt ($${X}_{3})$$. The relevant properties of the cation have been taken into consideration for the development of the correlation because cations have a bigger impact than anions in the formulation of microemulsions according to literature^5,53^. All the features are dimensionless with respect to sodium chloride. The hydration thickness of cation ($${\Delta d}_{hyd}$$) is the difference between the bare ion radius ($${r}_{b})$$ and the hydrated radius of ion ($${r}_{h})$$. The bare ion radius is not included in the hydration term since it is included in the charge density parameter.

Table 1 shows the fundamental statistics of the three features which used to develop the MLR model. In this study, the dataset was randomly partitioned into two portions of training and testing dataset. The MLR model was developed using the training set (80% of total dataset), while the testing set (20% of total dataset) were utilized to evaluate the model's performance.

**Table 1:** Statistical description of input data that is used to develop the optimum salinity correlation.

	Charge density ratio of cation	Hydration thickness ratio of cation	Molecular weight ratio of salt
Min	0.46	0.74	0.91
Avg	3.26	1.12	2.23
Std	4.71	0.31	1.13
Var	22.23	0.094	1.27
Max	15	1.62	5.85

The following equation is developed using the MATLAB MLR tool:

7 $$\frac{{S}_{Salt}^{*}}{{S}_{NaCl}^{*}}=-10.8{X}_{1}+16.74{X}_{2}-1.92{X}_{3}+8.45{X}_{1}{X}_{2}-0.386{X}_{1}{X}_{3}+1.93{X}_{2}{X}_{3}-0.14{X}_{1}^{2}-10{X}_{2}^{2}+0.62{X}_{3}^{2}-3.95$$

where $${X}_{1}$$, $${X}_{2}$$, $${X}_{3}$$ are:

$${X}_{1}= \frac{{\rho }_{cation\, from\, salt}}{{\rho }_{sodium\, ion}} {X}_{2}=\frac{{\Delta d}_{{hyd}_{cation\, from\, salt}}}{{\Delta d}_{{hyd}_{sodium\, ion}}} {X}_{3}=\frac{{M}_{{W}_{Salt}}}{{M}_{{W}_{NaCl}}}$$

**Figure 4:**
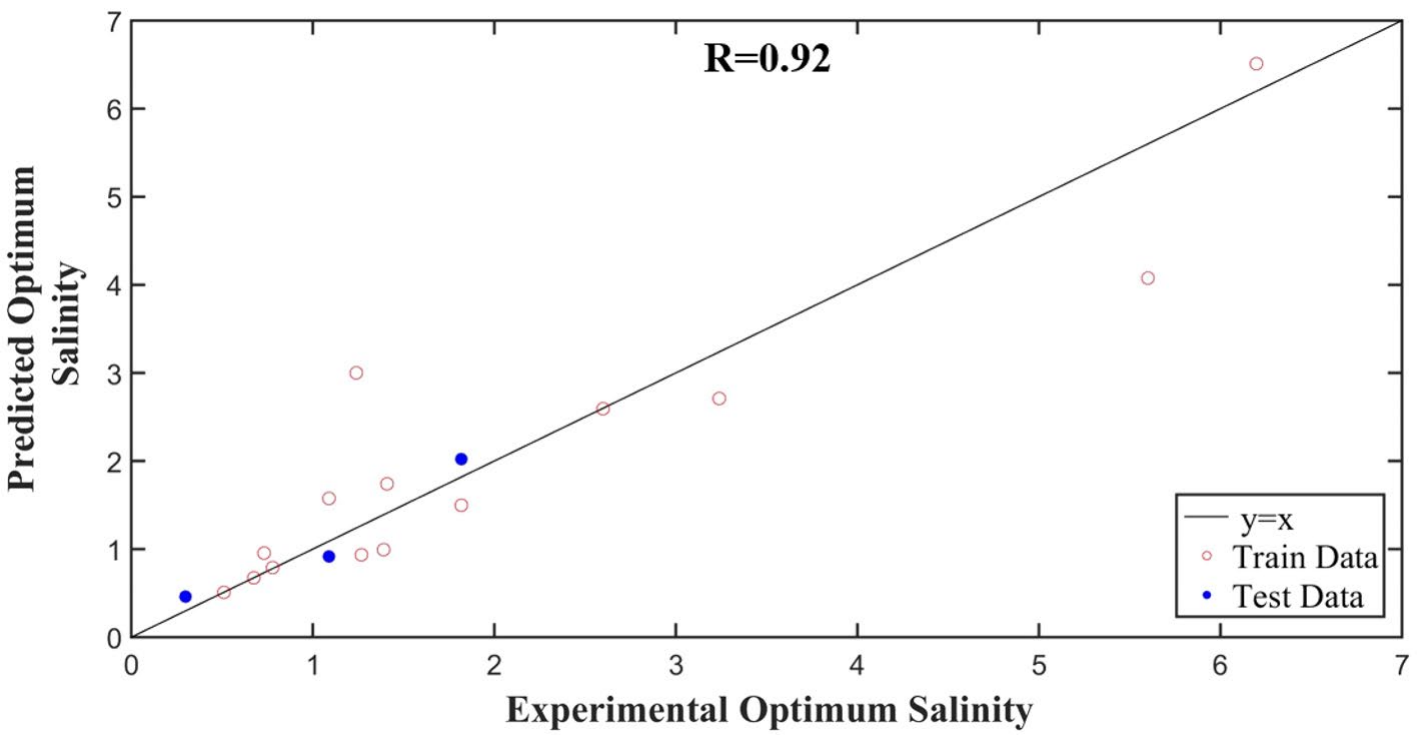
Cross plot between experimental and predicted optimum salinity of different salts by the proposed MLR model.

Figure 4 represents the cross plot to compare the MLR predicted data versus experimental data for training and testing dataset. The predicted optimum salinity is shown on the vertical axis of this figure, while the experimental optimum salinity of different salts is demonstrated on horizontal axis. Since almost all of the points from either the train or test dataset are located on the adjacency of y = x line, it can be inferred that the predicted and experimental data coincide well. The correlation coefficient of testing dataset is 92%, which reveals the precision of the model. It should be noted that this correlation has been developed for the optimum salinity data of available data including anionic surfactant and the sodium salts (different anions with sodium as cation) and chloride salts (different cations with chloride as anion). As a result, it can be expected that this correlation provides a more accurate prediction for these two types of salts. When the optimum salinity data for other types of salts are published, the credibility of the model can be assessed for other types of salts as well, and the model can even be improved.

The proposed correlation not only is appropriate for optimum salinity estimation of different salts but also is applicable for predicting the related salinities of Winsor I (${S}_{\mathrm{I}}$), Winsor II (${S}_{\mathrm{II}}$) as well as salinity values of phase transition points (${S}_{\mathrm{I}-\mathrm{III}}$, ${S}_{\mathrm{III}-\mathrm{II}}$).

### Effect of salts on NAC parameters

Taking into account the effect of other salts beside NaCl in the NAC equation could improve the prediction capability of the equation. To do so, the influence of salts on the parameters of this model must first be identified. So, we evaluate the length parameter (L) and characteristic length ($\xi$) in the presence of various salts by offering the corresponding correlations.

#### Effect of salts on surfactant length parameter

**Figure 5:**
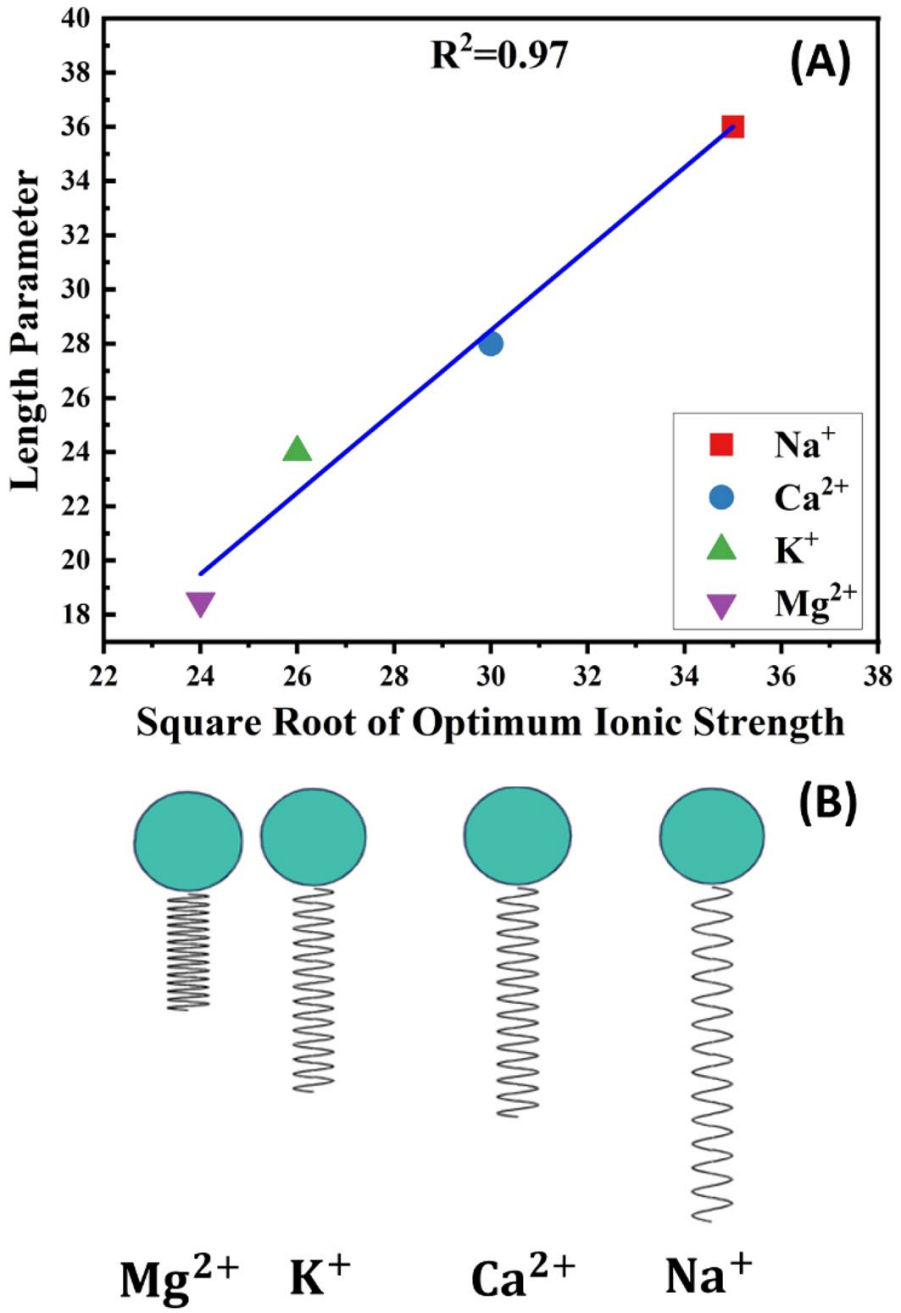
(**A**) Length parameter of SDBS vs. the square root of optimum ionic strength of different cations*.* (**B**) The compression-extension illustration of SDBS tail in presence of corresponding cations.

**Figure 6:**
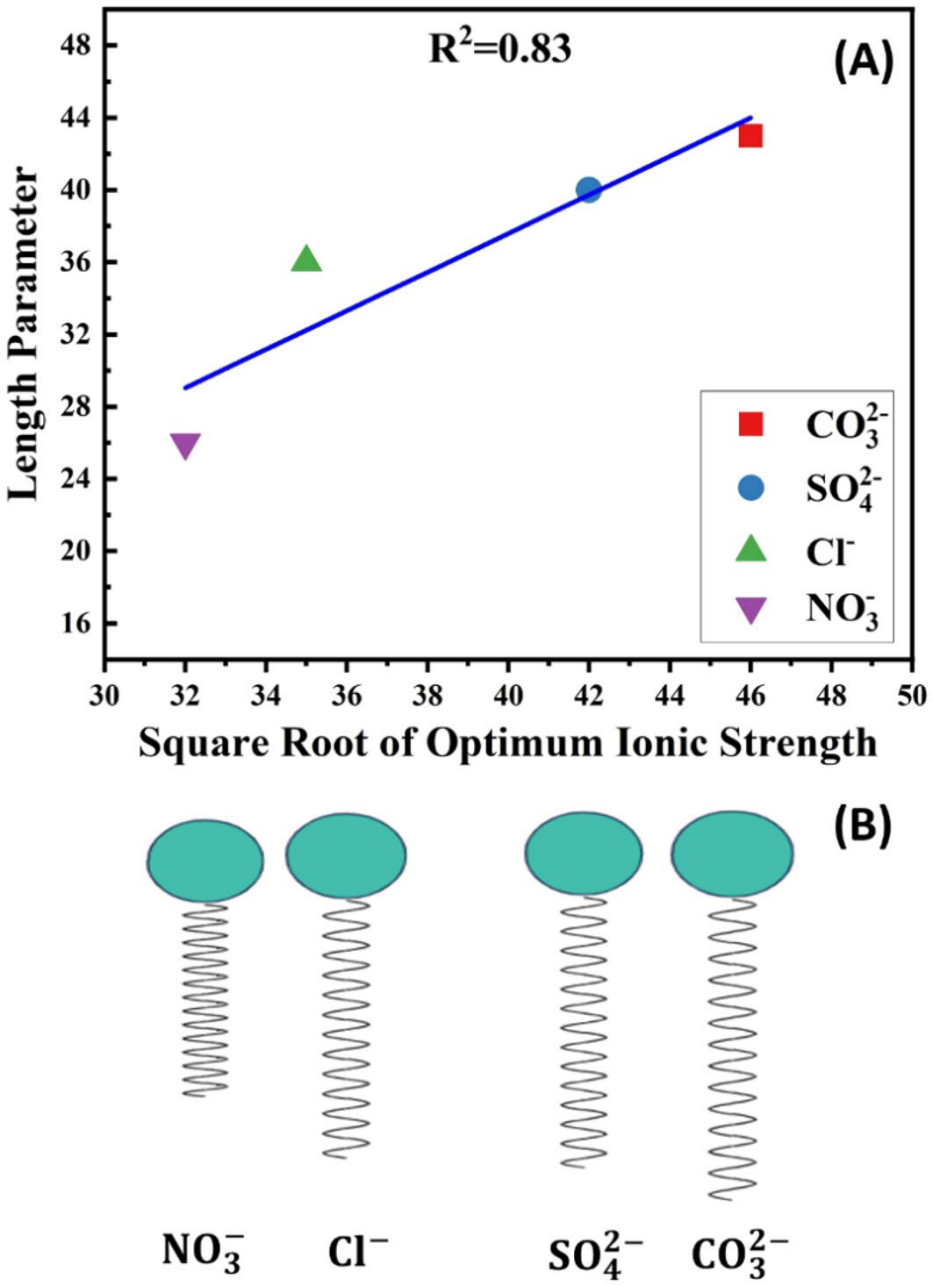
(**A**) Length parameter of SDBS vs. the square root of optimum ionic strength of different anions*.* (**B**) The compression-extension illustration of SDBS tail in presence of corresponding anions.

Here, we have investigated the influence of various salts on the surfactant’s length parameter. In this regard, we have fitted the SDBS length parameter in the SOW samples, which contain different salts using the experimentally measured phase volumes. The supplementary file S1 provides more information on how to fit length parameter from salinity scan data. The fitted L values versus the corresponding ionic strength of brine solutions at optimum salinity is shown in Figs. 5A and 6A for cations and anions, respectively. As can be seen from these figures the length parameter is linearly dependent on the brines’ optimum ionic strength. The high correlation factor (0.97 for cations and 0.83 for anions) is indicative of strong relationship between Length parameter and the optimum ionic strength. This means that the higher the optimum ionic strength of salt, the higher the length parameter of the surfactant**.**

This result can be interpreted in terms of "double-layer thickness”. According to Acosta, changes in the net-curvature (${H}_{n}$) of ionic surfactant films at oil–water interface is due to the changes in double-layer thickness. Double layer thickness determines the range of distance from the interface where the droplet surface potential vanishes and is reversely related to the ionic strength^54^:

8 $${\kappa }^{-1}=\frac{9.587* {10}^{-9}}{\sqrt{I}}$$

where ${\kappa }^{-1}$ and I are the double layer thickness (in nm) and ionic strength of salt (in $mol/{m}^{3}$) respectively. The net-curvature, on the other hand, has an inverse proportionality with the surfactant length parameter (according to Eq. 4). As a result, the length parameter and ionic strength are directly related. This explanation justifies the observed linear relationship between the length parameter and the optimum ionic strength^55,56^.

Generally speaking, since the salt nature has not been investigated enough in the previous studies, the importance of L is neglected. Therefore, the surfactant length scaling parameter has been only attributed to the number of carbon atoms in the surfactant tail chain. Here we illustrate that the surfactants in SOW systems containing salts with higher optimum ionic strength show higher length parameter and the opposite occurs for salts with the lower optimum ionic strength. It seems that this behavior illustrates the extension/compression of the surfactants tail in presence of different salts. Figures 5B and 6B illustrates the surfactant chain length with such an extension/compression behavior in the presence of different ions. As shown in these figures, the surfactant chain stretches in the presence salt with higher ${\mathrm{I}}^{*}$ but compresses in the presence of salts with lower ${\mathrm{I}}^{*}$. This is an indicator of variation of spring-like interaction of surfactant tail in the presence of different salts^57^.

#### Effect of salts on the characteristic length

**Figure 7:**
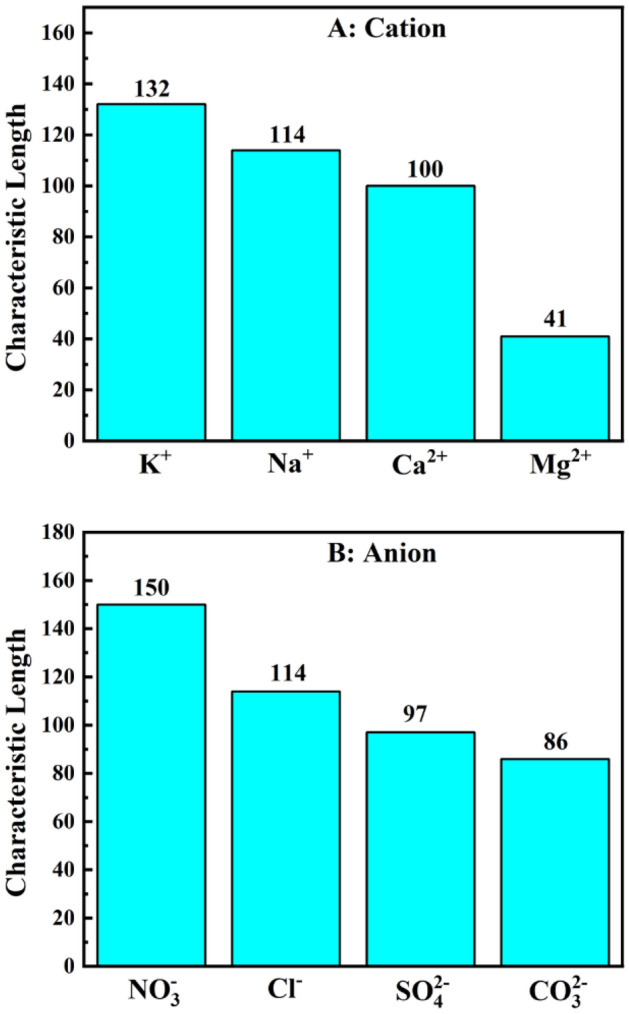
Effect of ions on the characteristic length of SDBS-hexane-electrolyte system. (**A**) Effect of cations from chloride salts. (**B**) Effect of anions from sodium salts.

As previously stated, in this study, both $\xi$ and L are fitted by minimizing the difference between the measured and calculated phase volume by performing the salinity scan. More details on fitting characteristic length from salinity scan data can be found in the supplementary file S1. In Fig. 7, the variation of characteristic length with the salt type is represented. As can be seen, the overall trend coincides with the Hofmeister series. In other words, the salting-out ions shrink the characteristic length, whereas the salting-in ions expand it. Salting-out ions reduce the water-solubility of the surfactant head, which is equal to decreasing the surfactant-water interaction. At the optimum condition, surfactant has the same affinity for the oil and water phases. Therefore, to maintain the equality of interactions there, surfactant-oil interaction must also be reduced^32^. Subsequently, the surfactant will have less control over the bulk molecules, reducing the characteristic length.

Previous studies confirm the obtained trend implicitly. It was seen before that changing the type of surfactant with various t head area affects the characteristic length of the system. They showed that the characteristic length decreases as the surfactant head area increases^58^. On the other hand, Vera et. al. have reported that in the presence of salting-out ions, the head-area of SDBS increases while in the presence of salting-in ions, it decreases^5^. As a result, our finding that the characteristic length decreases in the presence of salting-out ions and increases in the presence of salting-in ions is consistent with literature.

Moreover, the obtained trend for the effect of salt on the characteristic length follows the similar trend for the optimum solubilization parameter data on Fig. 2, which is representative of direct relationship between $\xi$ and ${SP}^{*}$ in accordance with the literature^4,19^.

## Conclusion

This work investigated how the type of salt affects the microemulsion formulation parameters and enhances the performance of HLD-NAC by proposing the salt impact on the length parameter and characteristic length, two key NAC parameters, for different salts. Our systematic study using HLD and HLD-NAC was consistent with the Hofmeister series, which showed that salting-out ions had lower optimal salinity and solubilization capacity than salting-in ions. It was shown that salt nature affects both the characteristic length and length parameter. The salts with the higher optimum ionic strength, expand the surfactant length parameter, whereas the salts with the lower optimum ionic strength, compress it. This is a sign of spring-like behavior of the surfactant tail when various salts are present. In terms of characteristic length, we found that the salting-out ions shrink the characteristic length while the salting-in ions do the reverse. Moreover, the MLR model was developed to estimate the optimal salinity of various salts based on more accessible parameters than previous studies. The correlation coefficient of 92.41% for the testing dataset demonstrated that the MLR model had a high prediction ability. We believe that our proposed model with a high degree of accuracy, will help researchers to design the emulsion systems more precisely.

### Supplementary Information

Supplementary Information.

## Data Availability

All data generated or analyzed during this study are included in this published article [and its supplementary information files].
