# Supplementary material for: An in-depth investigation of the impact of salt nature on the formulation of microemulsion systems
Source: Sci Rep. 2023 Sep 1;13:14362. doi: 10.1038/s41598-023-40761-x (PMC10474266; doi:10.1038/s41598-023-40761-x)
Supplement: Supplementary file 1 — Supplementary Information. [file 41598_2023_40761_MOESM1_ESM.docx]

Supplementary Information File for the article:

**An in-depth investigation of the impact of salt nature on the formulation of microemulsion systems**

${Ali Rezaie}^{1}$*,*${Hassan Ghasemi}^{2}$*,*${Fatemeh Eslami}^{3*}$

^1^Department of Chemical Engineering, Tarbiat Modares University, Tehran, Iran

^2^Department of Chemical Engineering, Tarbiat Modares University, Tehran, Iran

^3^Department of Chemical Engineering, Tarbiat Modares University, Tehran, Iran

**Corresponding Author:**

Fatemeh Eslami

Email: f_eslami@modares.ac.ir

**Content**:

This file includes 6 pages, four figures (Figures S.1–S.4), and one tables (Tables S.1). The following subjects are covered in the file:

***Salinity scans of SDBS-hexane-water systems in the presence of NaCl, MgCl_2_, NaNo_3_***

***The HLD-NAC correlations to calculate phase boundaries and fit L and*** $\xi$

***The prediction of phase volume fraction of SDBS-hexane-water systems in the presence of NaCl, MgCl_2_, NaNo_3_***

***Salinity scans of SDBS-hexane-water systems in the presence of NaCl, MgCl_2_, NaNo_3_***

All the tubes were placed at room temperature to reach their equilibrium states; around 24 hr. The optimal salinity state occurs when the three-phase state is formed with two excess aqueous and oil phases in the bottom and the top of tubes, respectively, which are approximately equal in volume.


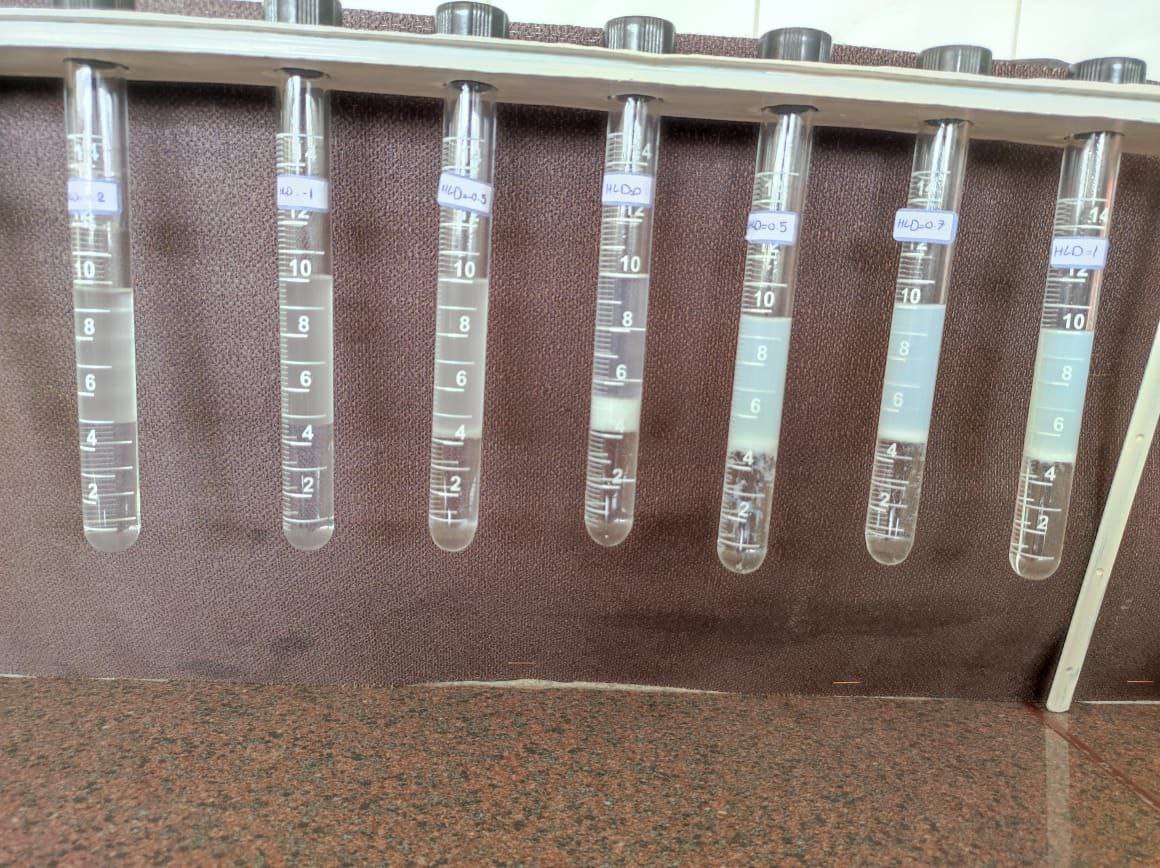


Figure S1. Salinity scan with NaCl as salt. HLD values range between -2 and +1. The optimal salinity is 6.8 g NaCl/ 100 ml aqueous solution


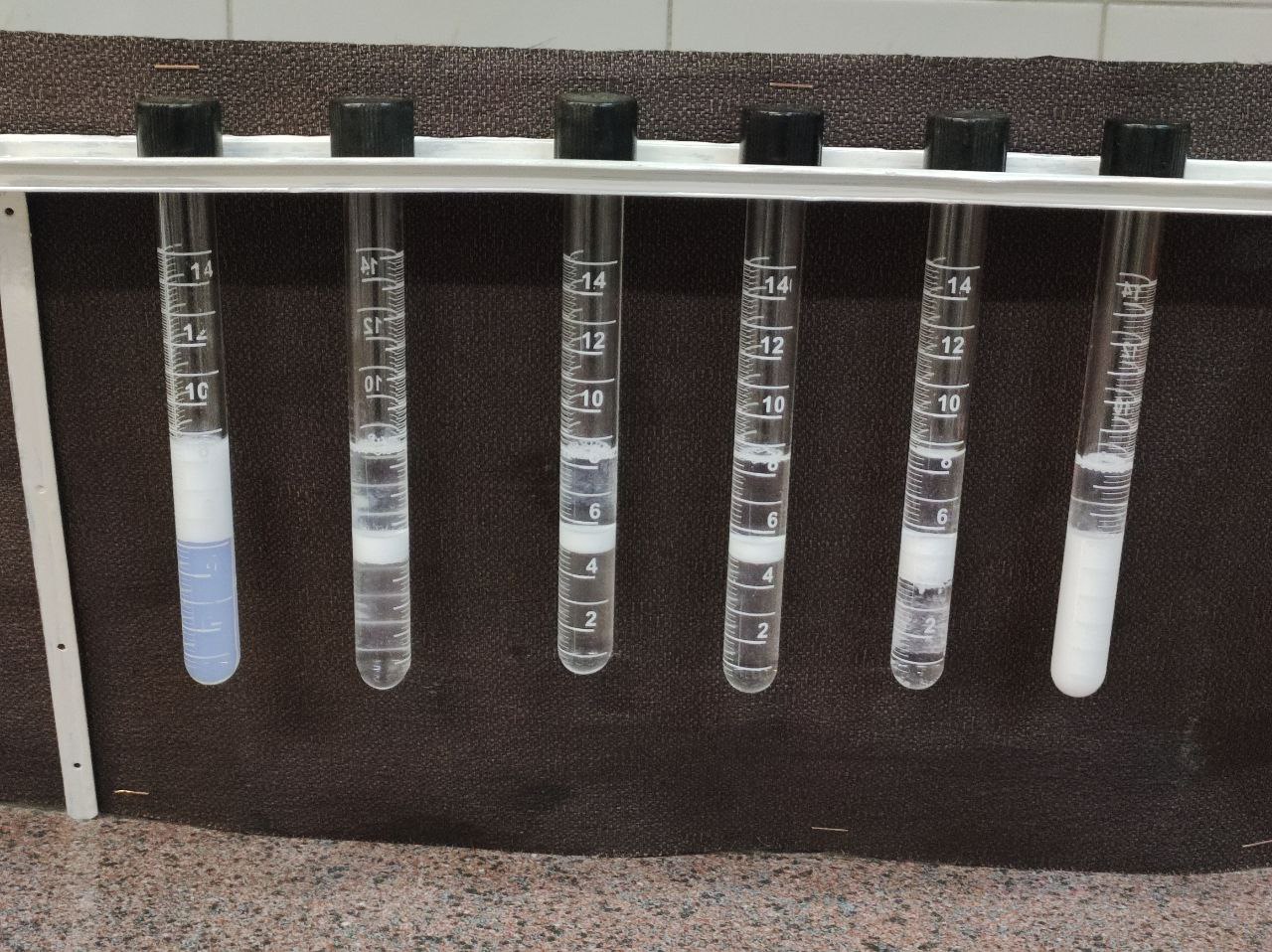


Figure S2. Salinity scan with MgCl2 with HLD values ranging between -1.2 and +1. The optimal salinity is 2 g ${MgCl}_{2}$/ 100 ml aqueous solution


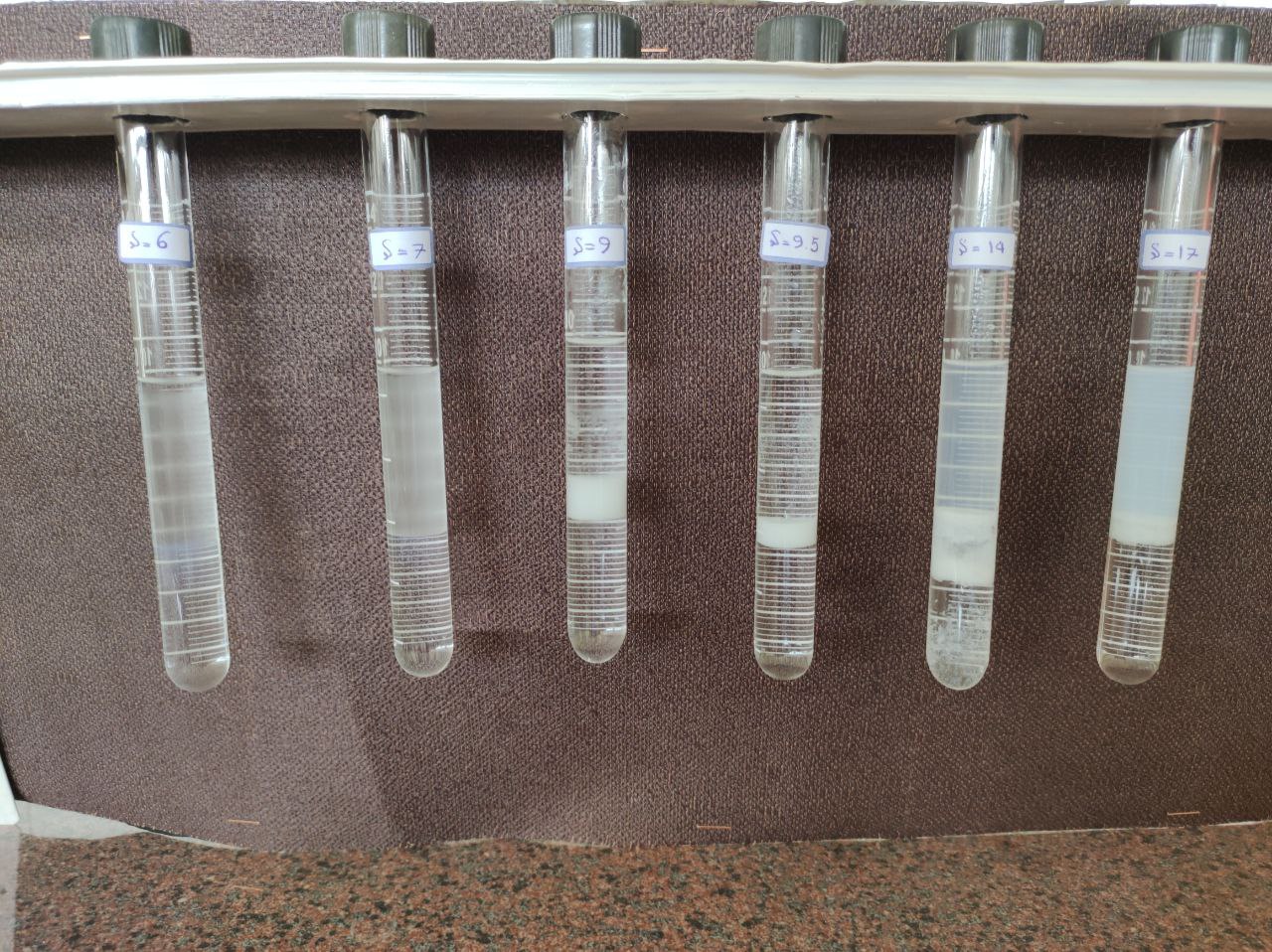


Figure S3. Salinity scan with NaNo3 with HLD values ranging between -0.45 and +0.6. The optimal salinity is 9.5 g ${NaNO}_{3}$/ 100 ml aqueous solution

***The HLD-NAC correlations to calculate phase boundaries and fit L and*** $\xi$

HLD-NAC can predict the properties of microemulsion systems using the L and $\xi$ and Upper (Ub) and lower (Lb) boundaries of phases in a salinity scan are one of these properties. In an equilibrated test tube, upper boundary (Ub) and lower boundary (Lb) are the interfaces between microemulsion and excess oil phase/excess water phases, respectively. In Winsor 1 systems, Lb is zero at the bottom of the tube. In Winsor 2 systems, Ub is one at the top of the tube. The calculation of upper and lower boundaries is as follows [1]:

| $Lb= \frac{R_{w, cont}- R_{w}}{R_{w,cont}+ R_{o,cont}}$ | (1) |
| --- | --- |
| $Ub= \frac{R_{w, cont}+ R_{o}}{R_{w,cont}+ R_{o,cont}}$ | (2) |

Where $R_{o}$ and $R_{w}$ represent the radii of coexisting hypothetical spherical aggregates of oil and water in the microemulsion phase. The calculation of hypothetical radii depends on the Winsor type of system. For type I (O/W) microemulsions, radius of the continuous aqueous phase and dispersed oil phase is calculated as follows:

| $R_{w}= R_{w,cont}= \frac{3 \times V_{w}}{A_{s}}$ | (3) |
| --- | --- |
| $A_{s}= \sum_{i} C_{s_{i}}\times V_{w} \times a_{i} \times6.023 \times{10}^{23}$ | (4) |
| $R_{o}= \frac{1}{\frac{1}{R_{w,cont}}- \frac{HLD}{L}}$ | (5) |

Where $V_{w}$ is the water volume in the system, $A_{s}$ represents total surfactant interfacial area, $C_{s_{i}}$ is concentration of surfactants in aqueous phase and $a_{i}$ is representative of surfactant head area per molecule.

For the case of type II (W/O) microemulsions, the calculations is as follows:

| $R_{o}= R_{o,cont}= \frac{3 \times V_{o}}{A_{s}}$ | (6) |
| --- | --- |
| $A_{s}= \sum_{i} C_{s_{i}}\times V_{o} \times a_{i} \times6.023 \times{10}^{23}$ | (7) |
| $R_{w}= \frac{1}{\frac{1}{R_{o,cont}}+ \frac{HLD}{L}}$ | (8) |

$R_{o}$ and $R_{w}$ are calculated in the following manner for type 3 microemulsions [2]:

| $R_{o}= \frac{1}{\frac{1}{\xi} - \frac{HLD}{2L}}$ |  | (9) |  |
| --- | --- | --- | --- |
| $R_{w}= \frac{1}{\frac{1}{\xi}+\frac{HLD}{2L}}$ |  | (10) | |

Having these Lb and Ub, we fit L and 𝜉 by the previously used procedure: [3]. In this regard, the following objective function is minimized:

| $objective function=\sqrt{\sum({(\mathrm{Ub}_{\mathrm{cal}}-\mathrm{Ub}_{\exp})}^{2}+{(\mathrm{Lb}_{\mathrm{cal}}-\mathrm{Lb}_{\exp})}^{2})}$ | (11) |
| --- | --- |

Regarding head surface area of SDBS as the surfactant in this work, we used salt-specific values as shown in Table S1. The data was gathered from a work by Forgiarini and co-workers[4].

Table S1. Surfactant head area values of SDBS as the surfactant in different saline conditions[4]

| **Surfactant Head Area (**${\boldsymbol{A^{\circ}}}^{\boldsymbol{2}}$**)** | **Salt** |
| --- | --- |
| 68 | No Salt |
| 57 | $KCl$ |
| 61 | $NaCl$ |
| 64.9 | ${CaCl}_{2}$ |
| 65.8 | ${MgCl}_{2}$ |
| 61.1 | ${NaNO}_{3}$ |
| 61.5 | ${Na}_{2}{SO}_{4}$ |
| 61.7 | ${Na}_{2}{CO}_{3}$ |

***The predictions of phase volume fractions of SDBS-hexane-water systems in the presence of NaCl, MgCl_2_ , NaNo_3_***

Using the framework in the last section, the fully-predicted phase volumes of different SOW systems are calculated. The results are reasonably accurate and consistent with experimental data and are shown in FigS4. Circles and triangular represent the experimental data. Each experiment was conducted three times, and error bars were calculated accordingly. The acceptable level of error bars indicates the reliability of volume fractions.


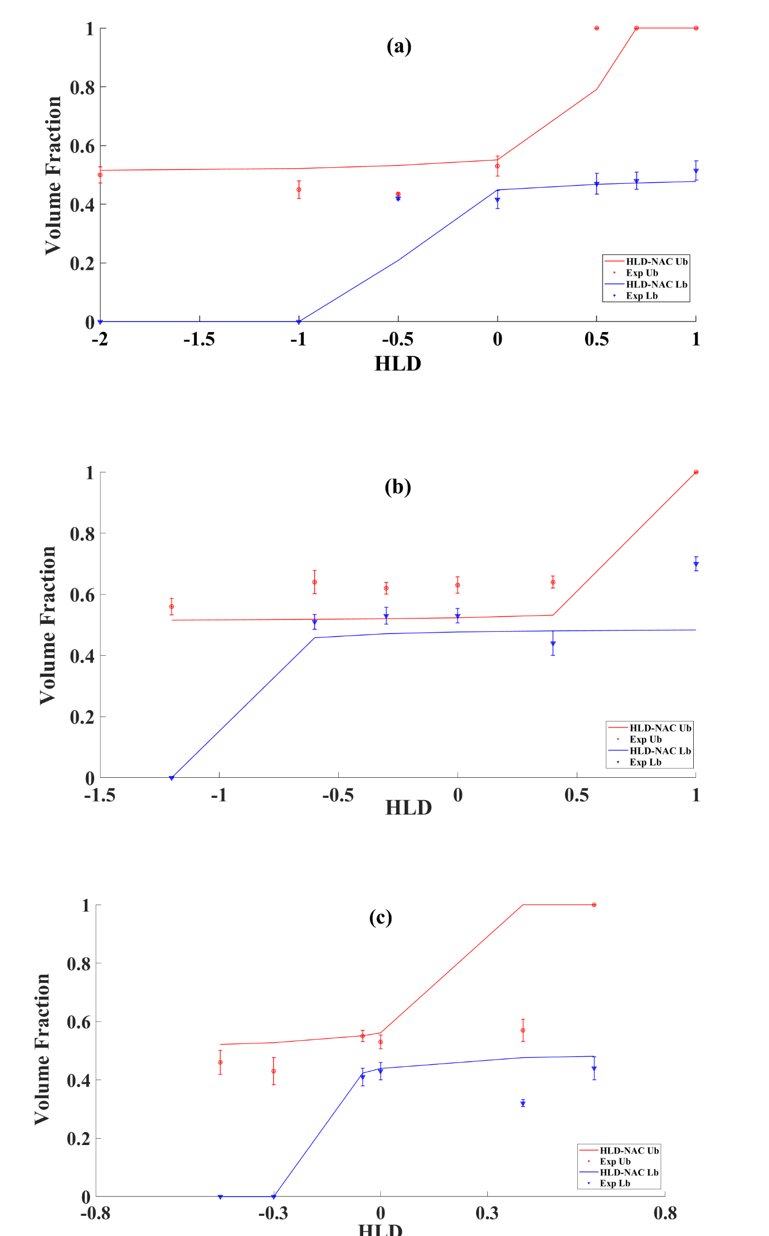


Figure S4. HLD-NAC prediction of Hexane-SDBS-water microemulsion system containing (a) NaCl, (b)MgCl_2_, (c) NaNo_3_ as salt. The solid lines are the HLD-NAC predicted values. After fitting HLD-NAC parameters to minimize the objective function, L = 36 and 𝜉 = 114 for system (a), L = 18 and 𝜉 = 41 for system (b), L = 26 and 𝜉 = 150 for system (c) were obtained.

**References**:

[1] EdgarAcosta, “Engineering cosmetics using the Net-Average-curvature (NAC) model,” *Curr. Opin. Colloid Interface Sci.*, vol. 48, pp. 149–167, 2020.

[2] Edgar Acosta, “The HLD–NAC equation of state for microemulsions formulated with nonionic alcohol ethoxylate and alkylphenol ethoxylate surfactants,” *Colloids Surfaces A Physicochem. Eng. Asp.*, vol. 320, no. 1–3, pp. 193–204, 2008.

[3] Hassan Ghasemi and Fatemeh Eslami, “Design of industrial wastewater demulsifier by HLD-NAC model,” *Sci. Rep.*, vol. 11, no. 1, pp. 1–12, 2021.

[4] R. E. Vera, F. Salazar-Rodríguez, R. Marquez, and A. M. Forgiarini, “How the Influence of Different Salts on Interfacial Properties of Surfactant–Oil–Water Systems at Optimum Formulation Matches the Hofmeister Series Ranking,” *J. Surfactants Deterg.*, vol. 23, no. 3, pp. 603–615, 2020, doi: 10.1002/jsde.12406.
